# A Secular Trend toward Earlier Male Sexual Maturity: Evidence from Shifting Ages of Male Young Adult Mortality

**DOI:** 10.1371/journal.pone.0014826

**Published:** 2011-08-17

**Authors:** Joshua R. Goldstein

**Affiliations:** Max Planck Institute for Demographic Research, Rostock, Germany; University of Turku, Finland

## Abstract

This paper shows new evidence of a steady long-term decline in age of male sexual maturity since at least the mid-eighteenth century. A method for measuring the timing of male maturity is developed based on the age at which male young adult mortality accelerates. The method is applied to mortality data from Sweden, Denmark, Norway, the United Kingdom, and Italy. The secular trend toward earlier male sexual maturity parallels the trend toward earlier menarche for females, suggesting that common environmental cues influence the speed of both males' and females' sexual maturation.

## Introduction

Improved nutrition and disease environments have generated substantial increases in human body size over recent centuries [1], [2]. A decline in the age of menarche, the measuring point for the onset of female sexual maturity, has also been well documented [3], [4]. A similar shift to earlier ages of sexual maturity for males has been hypothesized, but evidence of a long-term trend has been elusive [5], [6].

**Figure 1:**
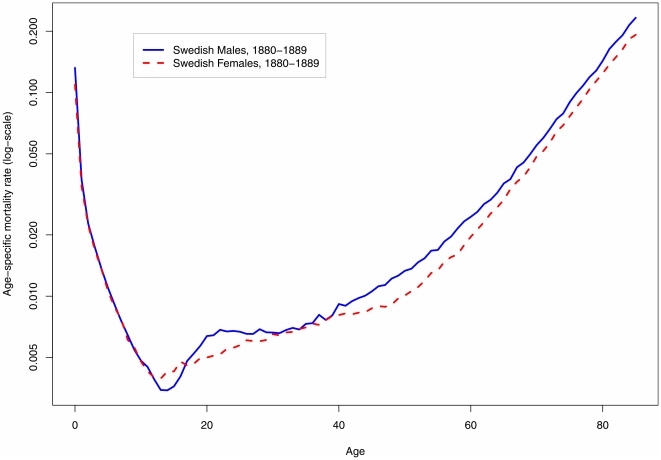
An example of the male ‘accident hump’ in illustrative logarithmic-scale age patterns of mortality in male and female humans. Source: Human Mortality Database [15].

The secular trend toward younger menarche can be documented because individual health records recording first menstruation can be compared over time. For males, however, no such comparable medical evidence exists. The research reported here takes an indirect approach to measuring males' age of sexual maturity. Nearly all human populations have exhibited a rise in mortality among males toward the end of adolescence. This rise, caused by increases in violent, accidental, and disease mortality, is known as the “accident hump.” Figure 1 shows an example of the presence of this hump for males (and its absence for females). The timing of surplus male mortality in early adulthood coincides broadly with peak male hormone production [7]. In this study, I use detailed historical mortality data to assess whether there has been a change over time in the age at which young male mortality peaks, and then hypothesize a correlation between earlier male mortality and earlier male sexual maturity.

Risk-taking and surplus mortality (the “accident hump”) are signatures of the male human's early adult years [8]. The main causes of death at these ages are accidents, violence, and disease [9]. Although the statistical influence of the accident hump on survival and life expectancy is small, the hump is visible relative to the low mortality typical of late adolescence and early adulthood, as Figure 1 demonstrates. (Interestingly, male non-human primates may also exhibit an accident hump [10], although small sample sizes make this claim conjectural [11], [12].) The accident hump does not occur in human females (or in female non-human primates, as far as is known).

The existence of a secular trend in the accident hump associated with male sexual maturity is of interest to developmental biology and biological life-history theory. The trend in female maturity is thought to be linked to improving nutrition and disease conditions, and may be a legacy of life-history plasticity in response to changing environments. Life-history theorists predict that better environmental conditions enable females to reproduce at younger ages [13]. Predictions for males are less clear but suggest a propensity toward increased somatic growth [14]. A secular trend toward earlier male maturity would suggest that similar environmental cues influence both males' and females' speed of maturation. Such a trend would demand a unified biological explanation for the pace of human development.

The accelerated pace of physical maturation among young adults would also be of interest to social scientists. Earlier physical maturity would contrast with the general trend toward later transition to adulthood in terms of social and economic roles.

## Methods

In order to analyze any change in the timing of the accident hump over time, I used high quality historical mortality estimates according to age that were produced by the Human Mortality Database. These estimates are available since 1751 in Sweden and since the mid-nineteenth century in Denmark, Norway, the United Kingdom, and Italy [15]. Age-specific mortality rates are calculated from registration records of deaths according to age and census estimates of the population according to age. I analyzed the data using cross-sectional period mortality comparisons among ages. In order to increase the robustness of estimates over time, I used 10-year periods (e.g., 1890–1899, 1900–1909, …) rather than single calendar years. Single-year age groups are used. Details of the estimation of mortality rates in the Human Mortality Database are available at [15]. (Cohort rates are analyzed in Appendix S1 and illustrated in Figure 4.)

**Figure 2:**
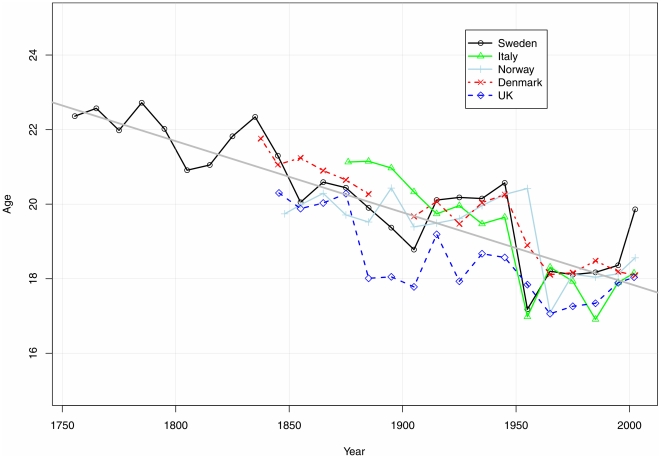
Estimated peak ages of male accident hump in various populations with high quality historical mortality records. Source: Author's estimates from period decadal abridged mortality rates available at Human Mortality Database [15]. The points are plotted at the middle of the decadal intervals. The slope of the fitted straight regression line is -0.19 years per decade.

The timing of the accident hump can be measured as the age at which surplus male mortality reaches its maximum. Because mortality does not necessarily fall after the hump, and in order to account for age-based senescent mortality, I took the maximum of the residuals obtained by fitting the Gompertz model of exponential mortality increase to older ages.

With M(x) denoting the mortality rate at age x, the Gompertz model is M(x)  =  a*exp(b*x), where a and b are estimated parameters. The Gompertz residual method fits a least-squares line to estimate “b,” the slope of the increase in the logarithm of mortality between ages 40 and 80. I then used the estimated regression equation to predict mortality from ages 10 to 40, and calculated residuals by subtracting the observed mortality at these ages. I estimated the peak of the accident hump by finding the age of largest residual mortality.

Non-integer ages of peaks were found by maximizing cubic-spline interpolated residual mortality between single years of age. In order to assure that the peak occurred at a “hump” and not at the youthful extreme of the ages considered, the local maximum for ages following any initial decline was taken as the accident hump. (In one case, Denmark 1890–1899, no such local maximum was available because residuals declined throughout the young-adult age range. The peak for this year was left unestimated.)

## Results

Figure 2 shows the trends in the Gompertz-residual measure of accident hump timing for England, Norway, Denmark, and Italy along with Sweden, all countries with accurate mortality time series available from the early- and mid-nineteenth century. For all countries, the timing of the accident hump fell steadily downward from the mid-eighteenth century to the mid-twentieth century, long before the introduction of the automobile in the twentieth century, and even before industrialization. Improved nutrition and disease environments, both of which have been shown to influence the production of testosterone [9], [16], [17], appear more plausible explanations for such long-term secular change than changing risk environment.

**Figure 3:**
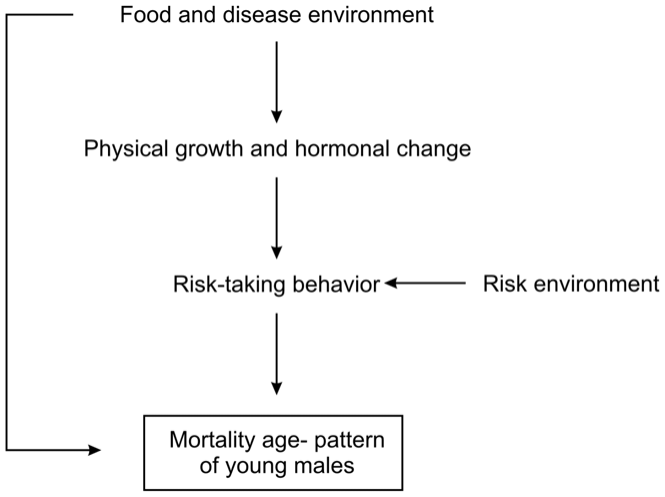
Causal diagram of selected hypothesized relationships influencing the male accident hump.

Figure 2 suggests that the secular trend toward earlier sexual maturity for males has halted in recent decades, as seems also to be the case with menarche [3], [18]. The implication that the age of sexual maturity in males has ceased to decline is consistent with modern measurements of males' age at puberty, as measured by testicular volume, which also suggest that little change in the timing of male sexual maturity has occurred in recent decades [19].

The average pace of decline of the age of sexual maturity for males between the mid-eighteenth century and the mid-twentieth century, about 0.2 years per decade, has been slightly slower than the 0.3 years per decade that menarche declined during the nineteenth and twentieth centuries [18].

## Discussion

The hypothesized causal relationships influencing the age patter of young male mortality are given in Figure 3. The mortality age-pattern is influenced by the timing of risk-taking behaviors. These behaviors are a function of the environment (e.g., when young men are allowed undertake dangerous activities like hunting or driving) and of physical and hormonal development. The food and disease environment influences the mortality age pattern directly through infectious diseases and indirectly by influencing growth and risk-taking behavior.

**Figure 4:**
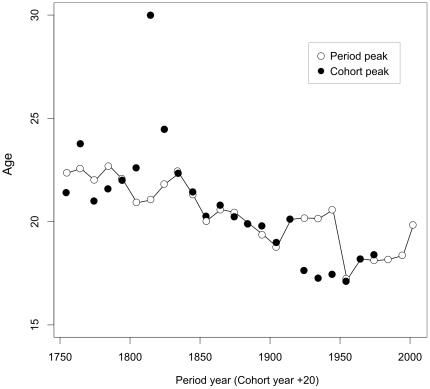
Estimated peak ages of male accident hump in Sweden from period and cohort mortality rates. Source: Source: Author's estimates from decadal abridged mortality rates available at Human Mortality Database [15]. The points are plotted in the middle of the decadal intervals. To make the two time series comparable, cohorts are plotted at the time the cohort reaches age 20.

Historical body heights are used to measure the nutritional status and disease environment of child populations [20]. If, as supposed here, the timing of male sexual maturity is also driven by nutritional status and disease environment, we would expect the secular trend in height to match the trend of the timing of the accident hump. However, the evidence here is not conclusive because of the largely monotonic trends in both the timing of the accident hump and in height. But what evidence can be found, as detailed below, suggests it is reasonable to attribute both phenomena to the same causes.

In England, the secular increase of heights was delayed compared to the other countries, which can reasonably be attributed to the consequences of urbanization and industrialization on disease environment and nutrition [21]. Large increases in height did not begin there until the end of the nineteenth century. Correspondingly, Figure 2 shows little change in the timing of the English accident hump until near the end of the nineteenth century. Similarly, the decades of largest height increases in Norway in the twentieth century coincide with a shift in the accident hump following World War II [22].

An additional piece of evidence in favor of a biological explanation for the secular trend in the accident hump is that another correlate of male sexual maturity, age at voice change, has also shown secular change. Daw [23] reports that age at voice change in the boys' choir lead by J.S. Bach in Leipzig in the mid-eighteenth century averaged around 18 years, but that in twentieth century London age at voice change was closer to 13 years. (Similarly, the age of voice change in Bach's choir rose during the worsening nutritional conditions of the War of the Austrian Succession.) The secular decline and the responsiveness to short-term changes in conditions tend to corroborate the plasticity of the timing of male sexual maturity.

The slightly slower rate of decline in age of male maturity, when compared with the rate of decline of age of female maturity, is consistent with the postulation of life-history theory that female sexual maturity is more plastic than male sexual maturity [13]. According to this theory, good environmental conditions translate into earlier reproduction for females but increased somatic growth in males. Consistent with this theory, we might expect improved nutrition to accelerate female development more directly than male development.

Evolutionary explanations for the existence of the accident hump need to account for why a rise in mortality at ages of near-maximum reproductive value would not be strongly selected against. One possible explanation is that successful risk-taking may have led to higher status and higher fitness among males, resulting in increased access to females. Dominance over other males leads to reproductive success in some primates, such as baboons [24]. Perhaps risky behaviors such as hunting and fighting with other males offer reproductive payoff in terms of improved access to females. Gage [12] has noted, “It is curious that in humans this hump occurs not at the age of biological sexual maturity but at an older age, just prior to marriage, the age of social sexual maturity” (p. 200). An alternative evolutionary explanation is that risk-taking behavior may be an unselected-for by-product of some evolved fitness advantage. For example, the primary function of testosterone may be to build muscle mass, and risk-taking behavior may be only a side effect [14]. In this case, the evolutionary benefit of increased strength may outweigh, on average, the dangers of risk-taking behavior.

The secular trend in the accident hump does not allow us to choose between these competing explanations. The steady and gradual nature of the decline makes it likely that the change in male age at maturity was driven by the same forces that caused earlier menarche.

### Consequences

Some analysts see earlier menarche as an unmitigated social disaster [25] because it shortens childhood and increases sexual activity among girls. With earlier menarche come earlier coitarche and the attendant risks of pregnancy and sexually transmitted diseases [26].

Earlier male maturation, on the other hand, has not received the same attention. Earlier risktaking behavior among males may be dangerous because it occurs at an age when young men are less mentally and socially mature. On the other hand, younger ages may be safer ages for risktaking behavior, because parents tend to supervise their children more closely when the children are younger. The results shown here raise questions of whether the timing of other developmental processes, such as brain development, show a similar secular trend [27].

As noted earlier, the decreasing age of male sexual maturity runs counter to the delay in the social transition to adulthood that has been documented around the world [28], [29], [30]. The ages at which males marry, become fathers, complete school, begin their careers, and become financially and residentially independent from their parents have all moved to later, not earlier, ages over the past half-century or so. The result has been an enlargement of the period of life between adolescence and full adulthood and a distancing of many decisions from the recklessness of youth.

### Research Directions

Future research could examine changes in the historical tempo of physical growth from height records. Such studies are difficult, however, because of the restricted age range—e.g., ages of military conscription—for which height data are available. A second area that could be investigated is whether specific acts of risk-taking and/or violent behavior among males have tended to occur at earlier ages as the average age of sexual maturity has dropped. Historical crime statistics may be relevant. A third area that remains to be explored is whether the timing of male maturity influences the trajectory of male hormone production at older ages [31].

For biologists, the finding of a secular trend toward earlier male maturity and the implication that male and female maturation respond to the same environmental cues imply a need to formulate a unified biological explanation for the pace of human physical maturation.

## Supporting Information

Appendix S1 Cohort analysis.
(0.03 MB DOC)
