# Supplementary material for: A Secular Trend toward Earlier Male Sexual Maturity: Evidence from Shifting Ages of Male Young Adult Mortality
Source: PLoS One. 2011 Aug 17;6(8):e14826. doi: 10.1371/journal.pone.0014826 (PMC3157338; doi:10.1371/journal.pone.0014826)
Supplement: Appendix S1 — Cohort analysis. (0.03 MB DOC) [file pone.0014826.s001.doc]

**Appendix**

**Cohort Analysis**

In the body of the paper, using period mortality data, I presented results showing that the timing of the accident hump has shifted. Here I show that a cohort analysis of the same mortality data produces comparable results, with a steady long-term secular trend toward an earlier peak in the accident hump. The standard advantages of doing period analysis are that data is easily available, data quality within periods is comparable, and recent trends can be readily analyzed. When the period-to-period fluctuations in mortality are large relative to the cohort-to-cohort fluctuations, an additional advantage of period analysis, is that the age pattern will be a better indicator of the underlying effect of age. For example, if mortality rises at all ages in a particularly severe year, this overall increase could be misinterpreted as an age-specific effect when looking at cohorts. Period analysis alleviates this problem by, in effect, controlling for the background mortality conditions of the moment. These advantages aside, however, cohort analysis is a more direct method of studying the longitudinal development of mortality according to age.

Cohort mortality rates for a long time series are available for Sweden in the Human Mortality Data. I replicated the period analysis using single-year-of-age, 10-year cohort mortality rates.

The results of the Gompertz residual method are shown in Figure 4, which shows the dates for periods and, for comparability, the year in which each cohort turns age 20. In the cohort analysis, we see the same strong secular pattern toward earlier ages of peak surplus male mortality as we saw in the period analysis. The cohort of 1810-19 is an outlier, but otherwise the pattern observed for periods appears to be robust to the choice between period and cohort data.
